# Academic Anxiety in Spanish Higher Education: A Systematic Review

**DOI:** 10.3390/bs15020192

**Published:** 2025-02-11

**Authors:** Nahia Idoiaga-Mondragon, Mirari Gaztañaga, Ion Yarritu, Eider Pascual-Sagastizabal

**Affiliations:** 1 Department of Evolutionary and Educational Psychology, University of the Basque Country UPV/EHU, 48940 Leioa, Spain; ion.yarritu@ehu.eus; 2 Department of Basic Psychological Processes and Their Development, University of the Basque Country UPV/EHU, 20018 Donostia-San Sebastián, Spain; mirari.gaztanaga@ehu.eus (M.G.); eider.pascual@ehu.eus (E.P.-S.)

**Keywords:** academic anxiety, education, higher education, mental health, Spain, systematic review

## Abstract

Background: Anxiety disorders are among the most prevalent mental health issues of the 21st century, significantly impacting individuals and healthcare systems worldwide. Within higher education research, academic anxiety is particularly significant, as it encompasses the specific anxieties students face within academic environments, such as exams and public speaking. This study aims to provide a contemporary overview of academic anxiety within Spanish universities by addressing three key questions: (1) How has research on academic anxiety evolved in Spain? (2) What tools have been used to measure academic anxiety? (3) What factors and variables have been analyzed in relation to academic anxiety, and what are the main findings? Methods: A systematic review was conducted following PRISMA guidelines for study selection, data extraction, and synthesis. The analysis focused on PubMed, PsycINFO, and Web of Science databases, examining 25 eligible articles published before January 2023. The objective was to evaluate, organize, and synthesize the evidence presented in these articles. Results: The findings revealed that the majority of studies were conducted in the last decade, employing 20 distinct measurement tools and examining more than 40 associated variables. The academic anxieties investigated included various types such as test anxiety, language learning anxiety, math anxiety, public speaking anxiety, and discipline-specific anxieties like dissection or music performance anxiety. Additionally, the studies explored the relationships between these academic anxieties and other variables such as gender and age. Conclusions: The implications of these findings for education and potential avenues for future research are discussed.

## 1. Introduction

Anxiety disorders represent one of the most prevalent mental health challenges of the 21st century, imposing significant healthcare costs and contributing substantially to the global burden of disease (Bandelow & Michaelis, 2015). These disorders encompass a spectrum of conditions that disrupt emotional, physiological, and cognitive functioning. Within this broader framework, anxiety emerges in specific contexts, such as educational environments, where academic challenges frequently act as significant stressors.

In educational settings, certain situations, including examinations and public speaking, are commonly associated with heightened anxiety among learners. Academic anxiety, which occurs within the academic context, constitutes a distinct form of anxiety, differing from general categories such as state or trait anxiety (Horwitz et al., 1986; MacIntyre & Gardner, 1991; MacIntyre & MacDonald, 1998). Consequently, academic anxiety can manifest in various forms depending on the specific challenges faced by students. Cassady (2010) introduced the concept of “academic anxieties” to describe the diverse range of anxiety types experienced in educational settings. Among the various manifestations of academic anxieties are test anxiety (Cassady & Johnson, 2002; Pintrich & Schunk, 2002), foreign language anxiety (FLA; Casado & Dereshiwsky, 2001; Horwitz et al., 1986), math anxiety (Alexander & Martray, 1989; Wigfield & Meece, 1988), and public speaking anxiety (MacIntyre & Thivierge, 1995; Raja, 2017).

Foreign language anxiety, as a subtype, includes challenges like speaking anxiety, reading anxiety, and writing anxiety, all of which are influenced by fear of negative evaluation, low self-perceived proficiency, and communication apprehension (Horwitz et al., 1986; MacIntyre & Gardner, 1991). This form of anxiety can significantly hinder language acquisition and academic performance in multilingual or foreign language contexts (Zheng & Cheng, 2018). Additionally, Spain’s multilingual landscape, where regional languages such as Catalan, Basque, and Galician coexist alongside Spanish, may amplify foreign language anxiety due to heightened communication demands and sociolinguistic expectations.

Statistics anxiety, which specifically affects students in disciplines requiring quantitative analysis, is another significant subtype. It has been linked to avoidance behaviors, procrastination, and reduced academic performance (Onwuegbuzie et al., 2000). This anxiety often stems from a combination of low self-efficacy, fear of failure, and a perceived lack of competence in statistical methods, making it a critical area of focus for improving academic outcomes in quantitative fields.

Communication anxiety, often observed in public speaking or group presentation contexts, is closely associated with intrusive thoughts and physiological responses like increased heart rate or sweating (Vera et al., 2020). These symptoms can severely impact academic performance, especially in disciplines where oral presentations are an essential component. Addressing this form of anxiety is vital for fostering effective communication skills, which are increasingly valued in higher education and professional environments.

Academic anxiety is characterized by an array of emotional, physiological, behavioral, and cognitive responses triggered by specific academic situations (Horwitz et al., 1986; Pintrich & Schunk, 2002). Students may experience frustration or excessive worry in response to anxiety-provoking stimuli such as exams or oral presentations. Physical manifestations, including sweating, headaches, or palpitations, are also frequent (Amengual-Pizarro, 2019). Such responses can result in behavioral paralysis (Tobias & Weissbrod, 1980) or the avoidance of academic tasks perceived as intimidating (MacIntyre & Gardner, 1991). Additionally, academic anxiety is often accompanied by expectations of failure and self-doubt (Calvo & Carreiras, 1993). These intrusive thoughts often arise involuntarily, repeatedly, and with significant disruptive potential (Wigfield & Meece, 1988).

Intrusive thoughts have been identified as a critical factor contributing to the adverse impact of academic anxiety on performance (Sarason, 1986). While some studies suggest that anxiety might occasionally facilitate academic performance (Alpert & Haber, 1960; Spielmann & Radnofsky, 2001), the majority of research highlights an inverse relationship between anxiety levels and academic achievement (e.g., Cassady & Johnson, 2002; Sarason, 1986). MacIntyre et al. (1997) proposed that heightened anxiety in academic settings can initiate a feedback loop, leading to poor performance and further amplifying anxiety, thus perpetuating a harmful cycle (see also Andujar & Cruz-Martínez, 2020). Sarason (1986) suggested that intrusive worrying thoughts impair the allocation of attention to relevant academic tasks, resulting in diminished performance.

The profound influence of academic anxiety on performance and overall well-being underscores the necessity for comprehensive studies addressing this issue. In particular, there is a need for systematic investigations consolidating data on the prevalence of various academic anxieties across diverse educational settings. Recent studies, such as those by Silaj et al. (2021) and Kang and Moreau (2023), highlight the importance of innovative interventions and contextual adaptations to mitigate academic anxiety effectively. This systematic review seeks to bridge this gap by examining the incidence of academic anxiety within Spanish universities. Accordingly, this study synthesizes data from investigations into different forms of academic anxiety experienced by university students in Spain to date.

## 2. Materials and Methods

### 2.1. The Design

In this review, the Preferred Reporting Items for Systematic Reviews and Meta-Analyses (PRISMA) checklist and flowchart were followed to ensure the highest possible rigor of the data obtained (Page et al., 2021).

### 2.2. Search Strategy

The search was conducted on 18 January 2023 across three databases: PubMed, PsycINFO, and Web of Science. Boolean operators (e.g., AND, OR) were applied to refine the search strategy. The initial search terms included “Anxiety”, “University”, and “Spain”. To ensure comprehensive coverage, the initial search was intentionally broad and later refined to focus on academic anxiety by applying filters and reviewing retrieved articles. Duplicate records were identified and excluded using the RefWorks platform, ensuring that unique references were retained for screening. This review included studies published before 18 January 2023.

### 2.3. Eligibility Criteria

To ensure consistency and standardization during the screening process, the following inclusion and exclusion criteria were established:

Inclusion Criteria:

Studies were eligible for inclusion if they met all the following criteria:

- They were peer reviewed scientific articles.
- They were published in English or Spanish.
- The study sample consisted of university students in Spain.
- They employed validated measurement instruments to assess anxiety.
- They provided data specifically addressing academic anxiety in the Spanish university population.

Exclusion Criteria:

Studies were excluded if they met any of the following criteria:

- They focused on interventions or the validation of anxiety measurement instruments.
- They utilized non-validated instruments to measure anxiety.
- The study sample included individuals outside the Spanish university population (e.g., mixed populations or other educational levels).
- They were not peer reviewed articles, such as conference abstracts or book chapters.
- Full-text access to the study was not available.
- They did not specifically address academic anxiety.

Redundant criteria were consolidated to eliminate overlap and ensure clarity. For example, criteria related to language and sample characteristics were revised to avoid duplication, and consistent terminology (e.g., “study”) was applied throughout.

### 2.4. Identification of Relevant Evidence and Data Mining

**Figure 1:**
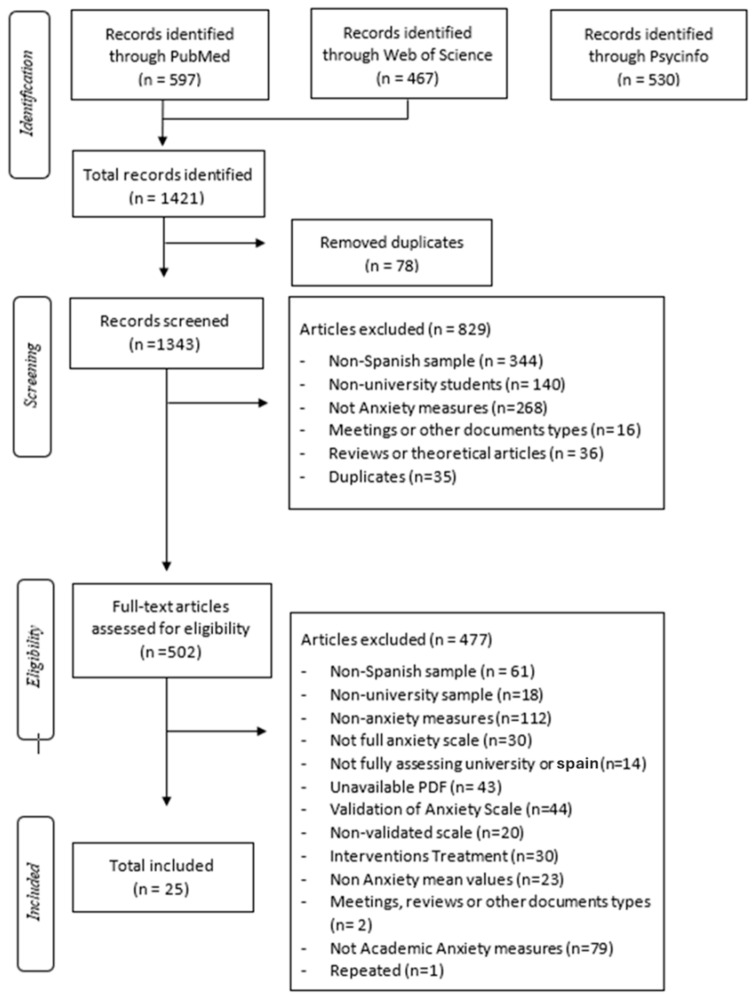
PRISMA flowchart of study selection.

The initial search retrieved 467 results from Web of Science, 597 from PubMed, and 530 from PsycINFO, yielding a total of 1421 documents. References were exported to RefWorks, where 78 duplicates were identified and removed. The remaining 1343 records underwent title and abstract screening by four independent reviewers. Based on the exclusion criteria, 829 documents were excluded, leaving 502 articles for full-text screening. This second stage of screening applied the additional inclusion criterion, “The study provides data on academic anxiety in the Spanish university population”, and the exclusion criterion, “The study investigates other types of anxiety”. Following this comprehensive process, 25 studies were selected for data extraction (see Figure 1).

Data extraction was performed by two researchers and included (a) authors and publication year, (b) sample size and characteristics, (c) measurement instruments for anxiety assessment, (d) other variables analyzed, and (e) reported findings on academic anxiety.

### 2.5. Assessment of Study Quality

The Quality Assessment Tool for Observational Cohort and Cross-Sectional Studies (National Heart, Lung, and Blood Institute, 2014) was used to evaluate the included studies. Key criteria assessed included the research question, study population, participation rate, inclusion and exclusion criteria, sample size justification, exposure and outcome measurement, blinding of assessors, follow-up processes, and control for confounding variables. Two authors independently evaluated each study, with discrepancies resolved through discussion or consultation with a third author when necessary.

## 3. Results

The results are discussed in terms of two main aspects: the elements used to categorize the 25 articles presented in Table 1 ((a) authors and year, (b) sample, (c) anxiety scale, (d) other analyzed variables, and (e) results) and (f) total quality assessment score based on Appendix A.

The findings of this systematic review offer a comprehensive perspective on academic anxiety in Spanish universities, presenting key insights into its various forms, associated factors, and contextual influences. The discussion integrates both the categorization of reviewed studies and their quality evaluation to provide a holistic understanding of the subject matter.

### 3.1. Year and Authors

The analysis of academic anxiety in Spanish colleges and universities has gained special relevance in recent decades, with most studies (19 out of 25) conducted since 2010. This surge reflects a growing recognition of the importance of mental health in academic contexts. The pioneering study by Calvo and Carreiras (1993) laid the groundwork for subsequent research, followed by a steady increase in publications during the 2000s and 2010s (e.g., Aguilar et al., 2006; Arnaiz-Castro & Guillén, 2013). Recent studies (2020 onwards) have further expanded the scope, incorporating innovative methodologies and diverse anxiety types (Andujar & Cruz-Martínez, 2020; Trigueros et al., 2020). Two key contributors, Arnaiz and Guillén and Arraez-Aybar, have provided consistent contributions to the field, emphasizing the role of contextual factors and demographic variables in shaping academic anxiety.

### 3.2. Anxiety Scale

A wide range of validated instruments were employed across the studies to measure academic anxiety, reflecting the complexity of this phenomenon. The State–Trait Anxiety Inventory (STAI) was the most frequently used tool, appearing in six studies (e.g., Arráez-Aybar et al., 2004; Núñez-Peña & Bono, 2019). Other commonly applied scales included the Foreign Language Classroom Anxiety Scale (used in five studies) and the Mathematics Anxiety Rating Scale (used in four studies). These tools facilitated the assessment of specific anxiety types, such as test anxiety, public speaking anxiety, and language anxiety, providing a nuanced understanding of their impact on students’ academic experiences.

### 3.3. Other Analyzed Variables

The reviewed studies examined a diverse array of variables influencing academic anxiety, with a focus on demographic factors (e.g., gender, age) and contextual elements (e.g., academic task complexity, assessment methods). Notably, gender differences were frequently highlighted, with female students reporting higher anxiety levels across several contexts, such as language learning and mathematics (Arnaiz & Guillén, 2012; Delgado-Monge et al., 2020). Furthermore, innovative pedagogical approaches, such as the use of the Escape Room method, demonstrated significant potential in reducing anxiety compared to traditional evaluation methods (Molina-Torres et al., 2021).

### 3.4. Results

The findings of the studies reviewed provide valuable insights into the multifaceted nature of academic anxiety in Spanish universities, linking various forms of anxiety to academic contexts and demographic factors. Below, the findings are synthesized into key themes to highlight interconnected patterns and their broader implications.

Academic anxiety was consistently associated with challenging academic tasks. For instance, test anxiety emerged as one of the most prevalent forms of academic anxiety. Higher levels were reported during assessments involving greater complexity or stakes (Aguilar et al., 2006). By contrast, innovative assessment methods, such as the Escape Room approach, demonstrated a reduction in anxiety compared to traditional evaluations (Molina-Torres et al., 2021), suggesting that pedagogical strategies can play a vital role in mitigating academic stress. The method of assessment, therefore, appears central to influencing anxiety levels.

Similarly, foreign language anxiety was identified as a significant issue, with key predictors including fear of negative evaluation, communication apprehension, and exam pressure (Amengual-Pizarro, 2019; Arnaiz & Guillén, 2012). Gender differences were notable, with female students reporting higher anxiety levels than males (Arnaiz & Guillén, 2012). The mandatory nature of language learning further intensified anxiety for students who perceived low proficiency or faced high expectations to succeed (Lahuerta, 2014). This highlights the importance of aligning language curricula with students’ abilities to alleviate anxiety.

Math anxiety disproportionately affected female students and those enrolled in health-related disciplines, while students in technical programs reported lower levels of anxiety (Delgado-Monge et al., 2020; Pérez-Tyteca et al., 2011). Furthermore, math-anxious students exhibited more intrusive thoughts and lower emotional regulation, which negatively impacted their working memory and performance in numerical tasks (Justicia-Galiano et al., 2016; Núñez-Peña & Bono, 2019). These findings emphasize the need for targeted support, particularly in fields where mathematics is perceived as a significant barrier to success.

Although less frequently studied, public speaking anxiety remains a critical concern. Intrusive thoughts and interpersonal anxiety were strongly linked to fear of public speaking, with physiological markers such as increased intraocular pressure observed during oral presentations (Vera et al., 2020; García Fernández et al., 2015). These findings underscore the importance of fostering confidence and providing training to reduce performance-related anxiety.

Among medical and nursing students, dissection anxiety revealed a gradual reduction with repeated exposure, though, initially, high levels persisted in some individuals (Arráez-Aybar et al., 2004, 2008). Emotional preparedness and attitudes toward life and death were identified as key factors influencing anxiety levels during dissection activities, highlighting the need for emotional support and preparatory interventions in these academic contexts.

Finally, broader cognitive and performance impacts of academic stress were evident in studies addressing reading anxiety and music performance anxiety. Anxious readers required greater cognitive effort to achieve comparable comprehension compared to non-anxious peers (Calvo & Carreiras, 1993). Similarly, solo music performers reported higher anxiety levels compared to ensemble performers, suggesting that the nature of academic tasks can significantly shape anxiety levels and coping strategies (Casanova et al., 2018).

### 3.5. Quality of Included Studies

Appendix A provides an assessment of the methodological quality of the included studies. All studies adequately defined their objectives, population characteristics, inclusion and exclusion criteria, the measured exposure, as well as the independent and dependent variables. However, none of the studies achieved 50% eligible participation or reported blinded outcome assessors, timeframe, or loss of follow-up information. Moreover, only one study (Trigueros et al., 2020) provided sample size justification, six studies assessed exposure more than once (Arráez-Aybar et al., 2004; Calvo & Carreiras, 1993; Grases et al., 2006; Martínez-Sánchez et al., 2003; Pelegrina et al., 2020; Vera et al., 2020), eight studies examined different levels of exposure (Aguilar et al., 2006; Arráez-Aybar et al., 2004; Calvo & Carreiras, 1993; Grases et al., 2006; Martínez-Sánchez et al., 2003; Molina-Torres et al., 2021; Pelegrina et al., 2020; Vera et al., 2020), and only three studies did not conduct statistical analyses where key potential confounding variables were measured (Arráez-Aybar et al., 2004; Arráez-Aybar et al., 2008; Caridad Ocerín et al., 2016).

## 4. Discussion

This systematic review has several noteworthy strengths in addressing one of the most prevalent mental disorders of the 21st century, anxiety (Bandelow & Michaelis, 2015), specifically focusing on academic anxiety in the context of higher education in Spain. Notably, to the best of our knowledge, this is the first research synthesis to analyze the incidence of academic anxiety within the Spanish university educational context. This review has made two key contributions. Firstly, it has enhanced our understanding of the extent to which this type of anxiety, which is directly related to educational practices, has been studied in Spain. Secondly, it has provided a comprehensive map of the current knowledge base on this topic.

The findings reveal that research on academic anxiety in Spanish colleges and universities has gained increased significance in recent decades, aligning with trends observed in other contexts (Silaj et al., 2021; Stearns, 2023). A plethora of authors have been actively contributing to this area of study, reflecting a growing concern within the scientific community (Kang & Moreau, 2023). However, the multitude of scales used to measure the construct (more than 20) and the wide range of variables analyzed (more than 40) pose challenges in extracting generalized results or conducting a meta-analysis, representing a limitation. Nevertheless, this systematic review followed the steps of research synthesis with scientific rigor (Cohen et al., 2018).

A critical finding is that academic anxiety has predominantly been studied in the contexts of exams, language learning, and mathematics. By contrast, less attention has been devoted to other domains, such as public speaking, medical studies, reading, and music-related degrees. This imbalance highlights potential areas for future research, particularly subgroup-specific analyses that could enable more targeted meta-analyses. Longitudinal studies would also be highly beneficial, as they could provide a dynamic understanding of how academic anxiety evolves across different stages of the academic journey. Expanding research to include group or individual work settings, private versus public universities, or various academic disciplines could offer valuable insights into the dynamics of academic anxiety (Silaj et al., 2021; Kang & Moreau, 2023).

The unique cultural and linguistic diversity of Spain adds an additional layer of complexity to academic anxiety. Multilingual demands in regions where Spanish coexists with languages like Catalan, Basque, and Galician may exacerbate foreign language anxiety (Zheng & Cheng, 2018). These findings underline the necessity for tailored interventions that consider regional and cultural contexts. Additionally, observed gender disparities in math anxiety emphasize the need to challenge societal stereotypes and implement targeted interventions through early educational reforms and support mechanisms (Delgado-Monge et al., 2020). Moreover, distinct subtypes of academic anxiety—such as communication anxiety, foreign language anxiety, and statistics anxiety—each have unique implications and require targeted strategies. For example, foreign language anxiety, often linked to fear of negative evaluation, significantly affects language learning outcomes (MacIntyre & Gardner, 1991). Similarly, statistics anxiety impacts performance in quantitative fields and necessitates specialized support mechanisms (Onwuegbuzie et al., 2000).

The implications of this review extend beyond academic research to practical applications in higher education policy and practice. Recognizing assessments as a significant source of anxiety, universities should adopt active learning methodologies such as gamified evaluations or collaborative assessments. These approaches have been shown to reduce test anxiety and foster engagement (Molina-Torres et al., 2021). Tailored language programs must address students’ proficiency levels while incorporating informal practice opportunities, such as peer-led groups or conversation workshops. Institutionalized stress management workshops could further equip students with proactive strategies to manage anxiety effectively.

Public speaking anxiety warrants particular attention, especially in academic programs where oral communication is critical. Structured, incremental training programs complemented by mindfulness techniques could significantly alleviate students’ fears and improve performance (Vera et al., 2020). Similarly, dissection anxiety in medical education could be mitigated through preparatory workshops and virtual simulations, easing students into hands-on experiences while building their confidence (Arráez-Aybar et al., 2004, 2008). Future research into underexplored domains such as reading and music-related anxieties could broaden our understanding of how academic anxiety manifests across diverse disciplines.

A noteworthy observation from this review is that academic anxiety has primarily been studied in relation to exams, language learning, and mathematics, with less emphasis on areas such as public speaking, medical studies, reading, and music-related degrees. This finding suggests numerous avenues for future research, including subgroup-specific analyses and meta-analyses focusing on specific domains. Extending the focus to encompass general anxiety and its prevalence among both students and university faculty could provide a more holistic understanding of anxiety within academic environments. Moreover, as all the studies reviewed here are cross-sectional, longitudinal research could offer valuable insights into how academic anxiety develops and changes over the course of students’ academic careers.

Conducting future meta-analyses on specific subtypes of anxiety could help unify fragmented data, providing a clearer picture of overarching trends. The recommendations provided aim to bridge the gap between research and practice, fostering an academic environment that prioritizes both educational success and student well-being.

## 5. Conclusions

This systematic review aimed to evaluate the current state of the literature on academic anxiety in Spanish higher education, identifying insights to guide strategies for addressing this issue. The findings highlight a predominant focus on individual contexts of academic anxiety, with a notable lack of studies exploring this phenomenon across different contexts or academic stages.

This review’s practical implications underscore the need for innovative pedagogical approaches to mitigate academic anxiety. Assessments, as a significant source of anxiety, can be improved through active learning methodologies like gamified evaluations and collaborative projects, which reduce stress and increase engagement (Idoiaga et al., 2024; Molina-Torres et al., 2021). Institutional workshops on stress management and exam strategies also equip students with tools to navigate high-pressure situations and enhance resilience.

Language learning anxiety, often tied to fear of communication and negative evaluation, requires tailored interventions. Structured language programs, informal conversation clubs, and peer mentoring networks provide safe, supportive spaces for students to build confidence and linguistic competence (Amengual-Pizarro, 2019; Dafouz, 2020). Similarly, mathematics anxiety could be addressed through inter-faculty tutoring programs and initiatives aimed at encouraging women’s participation in mathematics, given the higher anxiety levels observed among women and in feminized disciplines like health and education (Delgado-Monge et al., 2020; Pérez-Tyteca et al., 2011).

Public speaking anxiety, a common but under-researched issue, can be mitigated through incremental skill-building exercises, supported by mindfulness and relaxation techniques, to foster confidence and reduce physiological stress responses (Vera et al., 2020; García Fernández et al., 2015). In medical and nursing education, preparatory workshops and virtual dissection tools can help students manage dissection anxiety while addressing emotional and ethical aspects (Arráez-Aybar et al., 2004; Arráez-Aybar et al., 2008).

Finally, broader strategies are required to address cognitive and performance-related anxiety. Workshops on cognitive–behavioral techniques, such as reframing intrusive thoughts and enhancing working memory, can benefit students facing reading or performance anxiety. In music education, encouraging ensemble performances rather than solo activities can reduce pressure and foster collaboration (Calvo & Carreiras, 1993; Casanova et al., 2018).

The increasing prevalence of anxiety, especially among younger generations, demands institutional responsibility in addressing this issue. Academic anxiety is closely linked to pedagogical frameworks and institutional practices. Regular reviews of these practices are essential to minimize stress-inducing factors. By integrating innovative teaching and assessment methods that prioritize emotional well-being alongside academic rigor, universities can create supportive environments that promote both mental health and academic success. These measures emphasize the critical role of institutions in fostering a balanced and sustainable higher education system.

## Figures and Tables

**Table 1:** Analysis of the papers: (a) authors and year, (b) sample, (c) anxiety scale, (d) other analyzed variables, (e) results, and (f) total quality assessment score based on Appendix A.

Author(s) and Year	Sample	Anxiety Scale	Other Analyzed Variables	Results	Total Quality Assessment Score Based on Appendix A
(Aguilar et al., 2006)	192 students at the University of Valencia. 2 groups: (1) Total of 88 students (19 males and 69 females) aged between 22 and 59 years. (2) Total of 104 students (13 males and 91 females) aged between 20 and 40 years.	Anxiety and Performance Questionnaire 1	Age, gender. Personality Intelligence Motivation and an Attitude Questionnaire created ad hoc	The difficulty of the task is closely related to the variables analyzed, such as anxiety. Anxiety is a significant variable for participants who took the most difficult exam, while evaluation anxiety is not significant in the group with the easy exam.	8/13
(Amengual-Pizarro, 2019)	Total of 75 students from the University of Balearic Islands (63 females and 12 males) aged between 19 and 45 years.	Foreign Language Classroom Anxiety Scale 2	Gender, age, entry mark obtained in English. Formative itinerary or specialized education program.	Most foreign language students admitted to experiencing moderate anxiety when in class. However, 22.1% reported suffering from a high level of anxiety in classes. The data suggest that anxiety is primarily associated with the fear of communicating, negative evaluation, or exams.	7/13
(Andujar & Cruz-Martínez, 2020)	Total of 176 students from the University of Almeria (106 females and 79 males) aged between 18 and 64 years.	Cognitive Test Anxiety Scale-Revised 3 Structured Interview (designed by the Department of Psychology).	Gender, age	Moderate to high levels of cognitive test anxiety were found in the participants, which differed between two contexts: face-to-face examination and computer-based. Additionally, factors such as the absence of an examiner or not feeling observed during the speaking test performance were significant factors in determining anxiety levels.	7/13
(Arnaiz-Castro & Guillén, 2013)	Total of 200 students (109 females and 91 males) aged between18 and 39 years.	The Foreign Language Classroom Anxiety Scale 2	Gender, age, entry mark obtained in English, language level.	Anxiety is inherent in the learning process of foreign languages at the university level. Anxiety is lower in those students who study English as a main subject, compared with the participants who study it as a non-elective degree requirement. For the latter group, a stronger relationship was found between anxiety and the marks obtained by the participants.	7/13
(Arnaiz & Guillén, 2012)	Total of 216 students from the University of Las Palmas de Gran Canaria (120 females and 96 males) aged between 18 and 39 years.	The Foreign Language Classroom Anxiety Scale 2	Language level, gender, age, and grade obtained in the English language test.	Participants had an average level of anxiety, and females experienced more anxiety than males. Additionally, age showed a significant negative correlation with anxiety. Lower-grade students and lower-level students showed higher anxiety levels compared to the rest of the students.	7/13
(Arráez-Aybar et al., 2004)	Total of 399 students from the Complutense University of Madrid. Three studies. Study 1: Total of 92 participants (88% females and 12% males) aged between 17 and 29 years. Study 2: Total of 71 participants (88.7% females and 11.3% males) aged between 17 and 50 years. Study 3: Total of 236 participants (91.9% females and 8.1% males) aged between 17 and 40 years.	Study 1: State–Trait Anxiety Inventory (STAI) 4 Study 2: ISRA 5 and STAI 4 Study 3: STAI 4		The student’s anxiety response to the dissection is primarily determined by the characteristics of the situation. After being exposed to the situation, the anxiety response begins to decrease. However, the most anxious individuals tend to present higher anxiety levels than the less anxious individuals after several dissection sessions.	8/13
(Arráez-Aybar et al., 2008)	Total of 425 students from the Complutense University of Madrid (79.6% females and 20.4% males) with a mean age of 18 years.	Death Anxiety Inventory 6 Three instruments designed ad hoc about dissection: 1. Check-list of sensations experienced. 2. Pre-session. 3. Post-session questionnaire about attitudes and beliefs.		Participants showed a high level of anxiety before dissection. However, as they gained more experience of dissection, their emotional reactions were reduced, and their attitudes and beliefs changed. The level of death anxiety depends on the students’ perceptions of their degree of preparation for dissection, emotional control, and deeper thoughts about life and death during dissection.	6/13
(Calvo & Carreiras, 1993)	Thirty-six students. Two groups: (1) High anxiety group (*n* = 18). (2) Low anxiety group (*n* = 18).	State–Trait Anxiety Inventory (STAI) 4 Test Anxiety Inventory (TAI)	Four tests: two narrative and two expository created ad hoc. Reading times were recorded.	Anxious readers needed more processing resources than their non-anxious counterparts to obtain a similar comprehension level. Anxiety, therefore, is selectively detrimental to the efficiency of text-level processes. However, anxiety does not impair low-level processes such as encoding and lexical access.	9/13
(Caridad Ocerín et al., 2016)	Total of 120 students from the University of Huelva (82 females and 38 males) aged between 18 and 24.	Foreign Language Classroom Anxiety Scale 2		Students suffered from average to high anxiety levels. More specifically, students show high rates of anxiety in relation to communicative apprehension, a negative test evaluation, and fear of negative evaluation by the teacher and classmates.	6/13
(Casanova et al., 2018)	Total of 476 students from advanced musical learning institutions (252 males and 221 females) aged between 18 and 27: bowed strings (n = 140); woodwind instruments (n = 130); brass (n = 71); keyboard (n = 69); plucked strings (n = 31); singers (n = 19); percussionists (n = 13)	Kenny Music Performance Anxiety Inventory 8	Age, gender, academic year of enrollment, type of instrument, orchestra versus solo, and variables related to performance such as how frequently participants perform in ensembles.	A significant interaction was found between instrument type and academic year enrolment in the dimension of anxiety cognitions. Additionally, soloists showed higher levels of music performance anxiety during the four years of university-level studies compared to orchestra musicians.	7/13
(Delgado-Monge et al., 2020)	Total of 1190 students from the University of Granada (464 males and 726 females)	A subscale comprising 12 items developed by Fennema and Sherman (1976) to analyze anxiety for mathematics 15.	Gender and mathematical orientation of the studies.	The students with a greater quantity of mathematics courses showed mathematical anxiety, mathematical anxiety when facing problems, during exams, and in general. Female students showed higher anxiety levels than males.	7/13
(Fernández Rodríguez et al., 2019)	Total of 706 students from the University of Oviedo (492 females and 214 males) aged between 18 and 46 years	Hospital Anxiety and Depression Scale 9 Evaluation of the Need for Psychological Assistance Questionnaire 10	Gender, age, and other socio-demographic variables.	Academic conditions were the principal stressors, and no distinctive behavior profiles were found according to the branch of studies. Of the sample, 44.7% showed levels of anxiety and 13.5% depression. The fear of speaking in public, personal problems, distancing oneself from worries and emotions, and the promotion of healthy sleeping and eating habits were the areas of greatest demand for psychological support.	7/13
(García Fernández et al., 2015)	Total of 200 students (21.1% males and 78.9% females) aged between 18 and 30	Audience Anxiousness Scale 11 Rathus Assertiveness Schedule12 ISRA 5	Age, gender	A strong relationship was found between assertiveness and interpersonal anxiety and fear of public speaking.	7/13
(Grases et al., 2006)	Total of 35 students from the University of the Balearic Islands (12 males and 23 females) aged 18 to 20 years	State–Trait Anxiety Inventory 4	Urine samples for diuresis, pH, calcium, magnesium and creatinine. Perceived Stress Questionnaire 21	The students did not show increased stress during exams but suffered from higher anxiety. These results were compatible with the urinary biomarkers, such as a reduction in magnesium concentrations.	9/13
(Justicia-Galiano et al., 2016)	Total of 187 students from the University of Jaén (149 females and 38 males) aged 18 to 52	Revised Mathematics Anxiety Rating Scale 15 Statistical Anxiety Scale 16	White Bear Suppression Inventory 22 Cognitive Failures Questionnaire 23 Perceived Emotional Intelligence 24 Academic performance from “Data Analysis I” and “Socioemotional Development”.	Students with high math anxiety were more likely to experience intrusive thoughts, were less effective at suppressing these thoughts, and reported lower scores in understanding and regulating their emotions.	7/13
(Lahuerta, 2014)	Total of 195 students from the University of Oviedo. All were studying English for specific purposes as a subject	Communication Anxiety in English measured by 12 items for communication apprehension or anxiety.	Willingness to communicate in English Self-perceived Communication Competence in English 25 Motivation, Attitudes toward Learning English 26 Desire to learn English Attitude/Motivation Test Battery 27	The motivation to learn English was significantly related to their willingness to communicate in English. A relationship between self-perceived communication competence and willingness to communicate was also found, along with a negative relationship between anxiety and self-perceived communication competence.	7/13
(Martínez-Sánchez et al., 2003)	Total of 20 students from the University of Murcia aged 18 to 23 years.	State–Trait Anxiety Inventory 4	Pennebaker Inventory of Limbic Languidness 28 Toronto Alexithymia Scale 29	Emotional distress scores changed significantly depending on the phase (pre- or post-exam period). However, the level of alexithymia remained unchanged, showing that it represents a constant trait.	9/13
(Molina-Torres et al., 2021)	Total of 56 students	State–Trait Anxiety Inventory 4	Perceived Stress Questionnaire 24 Gaming Experience Scale 30	The levels of state anxiety and trait anxiety were higher in the traditional assessment group. The Escape Room strategy produces lower levels of anxiety and perceived stress during the evaluation.	8/13
(Nortes Martínez-Artero & Nortes Checa, 2014)	Total of 149 students (49% males and 51% females) aged from 17 to 28.	Mathematics Anxiety Rating Scale 15 “Anxiety” subscale from Attitude Scale 17	Gender, age, future professional career	A correlation between both scales was found. Participants with higher scores in mathematics anxiety showed more anxiety in the attitude scale. Women showed more anxiety than men; older students showed more anxiety than those under 21; and students that became mathematics teachers showed more anxiety than those who did not.	7/13
(Núñez-Peña & Bono, 2019)	Total of 180 s-year students enrolled on the “Research Design” course in the Psychology degree at the University of Barcelona (136 females and 44 males) aged 19 to 51.	Shortened math anxiety rating scale18 State–Trait Anxiety Inventory 4 German test anxiety inventory 19	A multiple-choice exam to evaluate the course.	Higher levels of math anxiety were related to low academic achievement, but a high level of test anxiety was related only to an increased number of errors. Women reported higher levels of trait, math, and test anxiety than their male peers, but their academic achievement was similar.	7/13
(Pelegrina et al., 2020)	Total of 114 psychology students (20 males and 94 females) aged 18 to 35.	Mathematics Anxiety Rating Scale 15	Working memory updating tasks.	A correlation was found between math anxiety scores and updating performance. Math-anxious individuals took longer and made more errors, especially on tasks that required retrieving information from working memory. Math-anxious individuals were less efficient in accessing numerical information in working memory.	9/13
(Pérez-Tyteca et al., 2011)	Total of 885 students from the University of Granada enrolled in mathematics, from various departments: Health (50), Experimental and Social Sciences (347), and Technical Education (339).	Mathematics Anxiety Rating Scale 15	Gender	Students showed anxiety levels below the neutral value. However, female participants showed significantly greater anxiety than men. Additionally, significant differences between fields of knowledge were found: students from technical degrees showed the lowest anxiety scores and students of health sciences the highest.	7/13
(Smyth et al., 2021)	Total of 394 students from Rey Juan Carlos University enrolled on a “Modern Language” course from a wide range of degrees such as Law, Education, Biology, and Engineering. Students were aged between 18 and 56.	Foreign Language Classroom Anxiety Scale 2 Test Anxiety Inventory (TAI) 7	Socio-demographic information (age, gender, and studies) Test of English for International Communication (TOEIC) Neo Five-Factor Inventory 31	Foreign Language Classroom Anxiety correlated most significantly with students’ foreign language academic performance, followed by the neuroticism dimension, test anxiety, and extraversion. These results show that anxiety can still be considered the best indicator to predict language academic performance and that personality traits do play a relevant role in the foreign language learning process in the university context.	7/13
(Trigueros et al., 2020)	Total of 1347 students belonging to the University of Almeria (733 males and 614 females) aged 19 to 27.	Test Anxiety Inventory (TAI) 7	Trait Meta Mood Scale 32 The Connor-Davidson Resilience Scale 33 The students Stress Inventory Stress Manifestations 34 Kidmed Scale to measure eating patterns 35	Emotional intelligence positively predicts resilience, whereas test anxiety and academic stress negatively predict resilience. Additionally, test anxiety and academic stress negatively predict the adherence to a Mediterranean diet. Therefore, academic transfer to university and grading pressure can generate maladaptive consequences for food consumption.	8/13
(Vera et al., 2020)	Total of 66 students from the final year of the degree in Optics and Optometry from the University of Granada.	Public Speaking Anxiety Scale 20	Stanford Sleepiness Scale 36 Intraocular Pressure Blood pressure	Higher intraocular pressure values were found before the oral presentation, which were positively associated with the perceived levels of public speaking anxiety. Therefore, intraocular pressure can be considered an indicator of stress in oral examination situations.	9/13

## Data Availability

The data presented in this study are available upon request from the corresponding author.

## Appendix A

**Table A1:** Quality of included studies.

Academic Anxiety Research	Q1	Q2	Q3	Q4	Q5	Q6	Q7	Q8	Q9	Q10	Q11	Q12	Q13	Q14	TOTAL YES
(Aguilar et al., 2006)	Yes	Yes	NR	Yes	No	Yes	No	Yes	Yes	No	Yes	No	NA	Yes	8/13
(Amengual-Pizarro, 2019)	Yes	Yes	NR	Yes	No	Yes	No	No	Yes	No	Yes	No	NA	Yes	7/13
(Andujar & Cruz-Martínez, 2020)	Yes	Yes	NR	Yes	No	Yes	No	No	Yes	No	Yes	No	NA	Yes	7/13
(Arnaiz-Castro & Guillén, 2013)	Yes	Yes	NR	Yes	No	Yes	No	No	Yes	No	Yes	No	NA	Yes	7/13
(Arnaiz & Guillén, 2012)	Yes	Yes	NR	Yes	No	Yes	No	No	Yes	No	Yes	No	NA	Yes	7/13
(Arráez-Aybar et al., 2004)	Yes	Yes	NR	Yes	No	Yes	No	Yes	Yes	Yes	Yes	No	No	No	8/13
(Arráez-Aybar et al., 2008)	Yes	Yes	NR	Yes	No	Yes	No	No	Yes	No	Yes	No	NA	No	6/13
(Calvo & Carreiras, 1993)	Yes	Yes	NR	Yes	No	Yes	No	Yes	Yes	Yes	Yes	No	No	Yes	9/13
(Caridad Ocerín et al., 2016)	Yes	Yes	NR	Yes	No	Yes	No	No	Yes	No	Yes	No	NA	No	6/13
(Casanova et al., 2018)	Yes	Yes	NR	Yes	No	Yes	No	No	Yes	No	Yes	No	NA	Yes	7/13
(Delgado-Monge et al., 2020)	Yes	Yes	NR	Yes	No	Yes	No	No	Yes	No	Yes	No	NA	Yes	7/13
(Fernández Rodríguez et al., 2019)	Yes	Yes	NR	Yes	No	Yes	No	No	Yes	No	Yes	No	NA	Yes	7/13
(García Fernández et al., 2015)	Yes	Yes	NR	Yes	No	Yes	No	No	Yes	No	Yes	No	NA	Yes	7/13
(Grases et al., 2006)	Yes	Yes	NR	Yes	No	Yes	No	Yes	Yes	Yes	Yes	No	NA	Yes	9/13
(Justicia-Galiano et al., 2016)	Yes	Yes	NR	Yes	No	Yes	No	No	Yes	No	Yes	No	NA	Yes	7/13
(Lahuerta, 2014)	Yes	Yes	NR	Yes	No	Yes	No	No	Yes	No	Yes	No	NA	Yes	7/13
(Martínez-Sánchez et al., 2003)	Yes	Yes	NR	Yes	No	Yes	No	Yes	Yes	Yes	Yes	No	NA	Yes	9/13
(Molina-Torres et al., 2021)	Yes	Yes	NR	Yes	No	Yes	No	Yes	Yes	No	Yes	No	NA	Yes	8/13
(Nortes Martínez-Artero & Nortes Checa, 2014)	Yes	Yes	NR	Yes	No	Yes	No	No	Yes	No	Yes	No	NA	Yes	7/13
(Núñez-Peña & Bono, 2019)	Yes	Yes	NR	Yes	No	Yes	No	No	Yes	No	Yes	No	NA	Yes	7/13
(Pelegrina et al., 2020)	Yes	Yes	NR	Yes	No	Yes	No	Yes	Yes	Yes	Yes	No	NA	Yes	9/13
(Pérez-Tyteca et al., 2011)	Yes	Yes	NR	Yes	No	Yes	No	No	Yes	No	Yes	No	NA	Yes	7/13
(Smyth et al., 2021)	Yes	Yes	NR	Yes	No	Yes	No	No	Yes	No	Yes	No	NA	Yes	7/13
(Trigueros et al., 2020)	Yes	Yes	NR	Yes	Yes	Yes	No	No	Yes	No	Yes	No	NA	Yes	8/13
(Vera et al., 2020)	Yes	Yes	NR	Yes	No	Yes	No	Yes	Yes	Yes	Yes	No	NA	Yes	9/13
TOTAL YES	100%	100%	0%	100%	8%	100%	0%	32%	100%	24%	100%	0%	0%	88%	

Q1 = clear objective; Q2 = clearly defined population; Q3 = 50% eligible participation; Q4 = inclusion and exclusion criteria of participants; Q5 = sample size justification; Q6 = exposure measured; Q7 = sufficient time frame; Q8 = different level of exposure examined; Q9 = clear independent variables; Q10 = exposure assessed more than once; Q11 = clear dependent variables; Q12 = outcome assessors blinded; Q13 = loss at follow-up; Q14 = confounding variables. NR, not reported. NA, not applicable.

## References

[B1-behavsci-15-00192] Aguilar A., Piera P. J. F., Aguilar E. M., Roldan C. (2006). El efecto de diferentes variables de personalidad sobre el resultado del examen realizado por dos muestras de estudiantes universitarios. Revista de Psicología General y Aplicada: Revista de la Federación Española de Asociaciones de Psicología.

[B2-behavsci-15-00192] Alexander L., Martray C. (1989). The development of an abbreviated version of the Mathematics Anxiety Rating Scale. Measurement and Evaluation in Counseling and Development.

[B3-behavsci-15-00192] Alpert R., Haber R. N. (1960). Anxiety in academic achievement situations. The Journal of Abnormal and Social Psychology.

[B4-behavsci-15-00192] Amengual-Pizarro M. (2019). Do prospective primary school teachers suffer from Foreign Language Anxiety (FLA) in Spain?. Vigo International Journal of Applied Linguistics.

[B5-behavsci-15-00192] Andujar A., Cruz-Martínez M. S. (2020). Cognitive test anxiety in high-stakes oral examinations: Face-to-face or computer-based?. Language Learning in Higher Education.

[B6-behavsci-15-00192] Arnaiz P., Guillén F. (2012). Foreign Language Anxiety in a Spanish University Setting: Interpersonal Differences//La ansiedad en el aprendizaje de una lengua extranjera en contexto universitario: Diferencias interpersonales. Revista de Psicodidáctica.

[B7-behavsci-15-00192] Arnaiz-Castro P., Guillén F. (2013). Anxiety in Spanish EFL students in different university degree programs. Anales de Psicología/Annals of Psychology.

[B8-behavsci-15-00192] Arráez-Aybar L. A., Casado-Morales M. I., Castaño-Collado G. (2004). Anxiety and dissection of the human cadaver: An unsolvable relationship?. The Anatomical Record Part B: The New Anatomist: An Official Publication of the American Association of Anatomists.

[B9-behavsci-15-00192] Arráez-Aybar L. A., Castaño-Collado G., Casado-Morales M. I. (2008). Dissection as a modulator of emotional attitudes and reactions of future health professionals. Medical Education.

[B10-behavsci-15-00192] Bandelow B., Michaelis S. (2015). Epidemiology of anxiety disorders in the 21st century. Dialogues in Clinical Neuroscience.

[B11-behavsci-15-00192] Calvo M. G., Carreiras M. (1993). Selective influence of test anxiety on reading processes. British Journal of Psychology.

[B12-behavsci-15-00192] Caridad Ocerin J., Holgado-Saez C., Mohamed Amar R. (2016). The influence of higher education contexts on levels of anxiety: A quantitative study of German. Profesorado-Revista de Curriculum y Formación de Profesorado.

[B13-behavsci-15-00192] Casado M. A., Dereshiwsky M. I. (2001). Foreign language anxiety of university students. College Student Journal.

[B14-behavsci-15-00192] Casanova O., Zarza F. J., Orejudo S. (2018). Differences in performance anxiety levels among advanced conservatory students in Spain, according to type of instrument and academic year of enrolment. Music Education Research.

[B15-behavsci-15-00192] Cassady J. C. (2010). Anxiety in schools. The causes, consequences, and solutions for academic anxieties.

[B16-behavsci-15-00192] Cassady J. C., Johnson R. E. (2002). Cognitive test anxiety and academic performance. Contemporary Educational Psychology.

[B17-behavsci-15-00192] Cohen L., Manion L., Morrison K. (2018). Research methods in education.

[B18-behavsci-15-00192] Dafouz E. (2020). Undergraduate student academic writing in English-medium higher education: Explorations through the ROAD-MAPPING lens. Journal of English for Academic Purposes.

[B19-behavsci-15-00192] Delgado-Monge I., Castro-Martínez E., Pérez-Tyteca P. (2020). Estudio comparativo sobre ansiedad matemática entre estudiantes de Costa Rica y España. Revista Electrónica Educare.

[B20-behavsci-15-00192] Fennema E., Sherman J. A. (1976). Fennema-Sherman mathematics attitudes scales: Instruments designed to measure attitudes toward the learning of mathematics by females and males. Journal for research in Mathematics Education.

[B21-behavsci-15-00192] Fernández Rodríguez C., Soto López T., Cuesta Izquierdo M. (2019). Needs and demands for psychological care in university students.

[B22-behavsci-15-00192] García Fernández C. M., Herruzo Cabrera F., Raya Trenas A. F. (2015). Temor a hablar en público en una muestra de estudiantes universitarios españoles. Ansiedad y estrés.

[B23-behavsci-15-00192] Grases G., Pérez-Castelló J. A., Sanchis P., Casero A., Perelló J., Isern B., Grases F. (2006). Anxiety and stress among science students. Study of calcium and magnesium alterations. Magnesium Research.

[B24-behavsci-15-00192] Horwitz E. K., Horwitz M. B., Cope J. (1986). Foreign language classroom anxiety. The Modern Language Journal.

[B25-behavsci-15-00192] Idoiaga N., Beloki N., Zarrazquin I., Artano K., Yarritu I. (2024). Active methodologies in Higher Education: Reasons to use them (or not) from the voices of faculty teaching staff.

[B26-behavsci-15-00192] Justicia-Galiano M. J., Pelegrina S., Lechuga M. T., Gutiérrez-Palma N., Martín-Puga E. M., Lendínez C. (2016). Math anxiety and its relationship to inhibitory abilities and perceived emotional intelligence. Anales De Psicología/Annals of Psychology.

[B27-behavsci-15-00192] Kang N. S., Moreau K. (2023). Excessive Evaluation Anxiety (XEA): The last two decades. Journal of MultiDisciplinary Evaluation.

[B28-behavsci-15-00192] Lahuerta A. C. (2014). Factors affecting willingness to communicate in a Spanish university context. International Journal of English Studies.

[B29-behavsci-15-00192] MacIntyre P. D., Gardner R. C. (1991). Language anxiety: Its relationship to other anxieties and to processing in native and second languages. Language Learning.

[B30-behavsci-15-00192] MacIntyre P. D., MacDonald J. (1998). Public speaking anxiety: Perceived competence and audience congeniality. Communication Education.

[B31-behavsci-15-00192] MacIntyre P. D., Noels K. A., Clément R. (1997). Biases in self-ratings of second language proficiency: The role of language anxiety. Language Learning.

[B32-behavsci-15-00192] MacIntyre P. D., Thivierge K. A. (1995). The effects of audience pleasantness, audience familiarity, and speaking contexts on public speaking anxiety and willingness to speak. Communication Quarterly.

[B33-behavsci-15-00192] Martínez-Sánchez F., Ato-García M., Ortiz-Soria B. (2003). Alexithymia—State or trait?. The Spanish Journal of Psychology.

[B34-behavsci-15-00192] Molina-Torres G., Sandoval-Hernández I., Ropero-Padilla C., Rodriguez-Arrastia M., Martínez-Cal J., Gonzalez-Sanchez M. (2021). Escape room vs. Traditional assessment in physiotherapy students’ anxiety, stress and gaming experience: A comparative study. International Journal of Environmental Research and Public Health.

[B35-behavsci-15-00192] National Heart, Lung, and Blood Institute (2014). Quality assessment tool for observational cohort and cross-sectional studies.

[B36-behavsci-15-00192] Nortes Martínez-Artero R., Nortes Checa A. (2014). ¿Tienen ansiedad hacia las matemáticas los futuros matemáticos? Profesorado. Revista de Currículum y Formación de Profesorado.

[B37-behavsci-15-00192] Núñez-Peña M. I., Bono R. (2019). Academic anxieties: Which type contributes the most to low achievement in methodological courses?. Educational Psychology.

[B38-behavsci-15-00192] Onwuegbuzie A. J., DaRos D., Ryan J. M. (2000). The components of statistics anxiety: A phenomenological study. Focus on Learning Problems in Mathematics.

[B39-behavsci-15-00192] Page M. J., McKenzie J. E., Bossuyt P. M., Boutron I., Hoffmann T. C., Mulrow C. D., Shamseer L., Tetzlaff J. M., Akl E. A., Brennan S. E., Chou R., Glanville J., Grimshaw J. M., Hróbjartsson A., Lalu M. M., Li T., Loder E. W., Mayo-Wilson E., McDonald S., Alonso-Fernandez S. (2021). PRISMA 2020 statement: An updated guideline for the publication of systematic reviews. Revista Española De Cardiología.

[B40-behavsci-15-00192] Pelegrina S., Justicia-Galiano M. J., Martín-Puga M. E., Linares R. (2020). Math anxiety and working memory updating: Difficulties in retrieving numerical information from working memory. Frontiers in Psychology.

[B41-behavsci-15-00192] Pérez-Tyteca P., Martínez E. C., Romero L. R., Martínez E. C. (2011). Ansiedad matemática, género y ramas de conocimiento en alumnos universitarios. Enseñanza de las Ciencias: Revista de Investigación y Experiencias Didácticas.

[B42-behavsci-15-00192] Pintrich P. R., Schunk D. H. (2002). Motivation in education: Theory, research, and applications.

[B43-behavsci-15-00192] Raja F. (2017). Anxiety level in students of public speaking: Causes and remedies. Journal of Education and Educational Development.

[B44-behavsci-15-00192] Sarason I. G., Schwarzer R. (1986). Test anxiety, worry, and cognitive interference. Self-related cognitions in anxiety and motivation.

[B45-behavsci-15-00192] Silaj K. M., Schwartz S. T., Siegel A. L., Castel A. D. (2021). Test anxiety and metacognitive performance in the classroom. Educational Psychology Review.

[B46-behavsci-15-00192] Smyth A. M., Manzanares N. G., Muñoz J. J. F. (2021). Anxiety and personality as indicators of academic performance in university foreign language classrooms. Porta Linguarum Revista Interuniversitaria de Didáctica de las Lenguas Extranjeras.

[B47-behavsci-15-00192] Spielmann G., Radnofsky M. L. (2001). Learning language under tension: New directions from a qualitative study. The Modern Language Journal.

[B48-behavsci-15-00192] Stearns P. N. (2023). Student anxiety and its impact: A recent american history. History of Education Quarterly.

[B49-behavsci-15-00192] Tobias S., Weissbrod C. (1980). Anxiety and mathematics: An update.

[B50-behavsci-15-00192] Trigueros R., Padilla A. M., Aguilar-Parra J. M., Rocamora P., Morales-Gázquez M. J., López-Liria R. (2020). The influence of emotional intelligence on resilience, test anxiety, academic stress and the Mediterranean diet. A study with university students. International Journal of Environmental Research and Public Health.

[B51-behavsci-15-00192] Vera J., Redondo B., Álvarez-Rodríguez M., Molina R., Jiménez R. (2020). The intraocular pressure responses to oral academic examination: The influence of perceived levels of public speaking anxiety. Applied Ergonomics.

[B52-behavsci-15-00192] Wigfield A., Meece J. L. (1988). Math anxiety in elementary and secondary school students. Journal of Educational Psychology.

[B53-behavsci-15-00192] Zheng Y., Cheng L. (2018). How does anxiety influence language performance? From the perspectives of Foreign Language classroom anxiety and cognitive test anxiety. Language Testing in Asia.

